# Machine Learning-Based Models for the Prediction of Postoperative Recurrence Risk in MVI-Negative HCC

**DOI:** 10.3390/biomedicines13102507

**Published:** 2025-10-15

**Authors:** Chendong Wang, Qunzhe Ding, Mingjie Liu, Rundong Liu, Qiang Zhang, Bixiang Zhang, Jia Song

**Affiliations:** 1 Hepatic Surgery Center, Tongji Hospital, Tongji Medical College, Huazhong University of Science and Technology, Wuhan 430030, China; m202376622@hust.edu.cn (C.W.); bixiangzhang@hust.edu.cn (B.Z.); 2 School of Information Management, Wuhan University, Wuhan 430072, China; dingqunzhe1@163.com; 3 Department of Oncology, Liyuan Hospital, Tongji Medical College, Huazhong University of Science and Technology, Wuhan 430077, China; m202376741@hust.edu.cn; 4 Department of Oncology, Tongji Hospital, Tongji Medical College, Huazhong University of Science and Technology, Wuhan 430030, China; m202476648@hust.edu.cn (R.L.); m202376633@hust.edu.cn (Q.Z.)

**Keywords:** hepatocellular carcinoma, machine learning, SHAP, early recurrence

## Abstract

**Background**: Hepatocellular carcinoma (HCC) patients without microvascular invasion (MVI) face significant postoperative early recurrence (ER) risks, yet prognostic determinants remain understudied. Existing models often rely on linear assumptions. This study aimed to develop and validate an interpretable machine learning model using routine clinical parameters to predict early recurrence (ER) in MVI-negative HCC patients. **Methods**: We retrospectively analyzed 578 MVI-negative HCC patients undergoing radical resection. Seven machine learning (ML) algorithms were systematically benchmarked using clinical/laboratory/imaging features optimized via recursive feature elimination (RFE) and hyperparameter tuning. Model interpretability was achieved via SHapley Additive exPlanations (SHAP). **Results**: The CatBoost model demonstrated superior performance (AUC: 0.7957, Accuracy: 0.7290). SHAP analysis identified key predictors: tumor capsule absence, elevated HBV-DNA and CA125 levels, larger tumor diameter, and lower body weight significantly increased ER risk. Individualized SHAP force plots enhanced clinical interpretability. **Conclusions**: The CatBoost model exhibits robust predictive performance for ER in MVI-negative HCC, offering a clinically interpretable tool for personalized risk stratification and optimization of postoperative management strategies.

## 1. Introduction

Hepatocellular carcinoma (HCC) is the most common form of primary liver cancer, accounting for 75–80% of all such malignancies [1]. Based on statistical data, approximately 905,700 new cases of liver cancer were diagnosed globally in 2020, and nearly 830,200 individuals succumbed to the disease [2]. Currently, radical hepatectomy is still the only curative treatment for HCC. Nonetheless, the clinical outcomes for HCC patients have been less than optimal, largely due to the high rate of postoperative recurrence. It is estimated that 50–70% of HCC patients experience relapse within five years after surgery [3]. Notably, up to 35% of HCC patients experience recurrence within 12 months postoperatively [4], representing a major determinant of survival outcomes. Clinically, HCC recurrence is categorized as early or late after hepatectomy. Early recurrence (ER), defined as recurrence within two years after liver resection (LR), is associated with significantly poorer prognoses compared to late recurrence [5].

Microvascular invasion (MVI), defined as the presence of microscopically visible cancer cell nests in endothelium-lined vessels and detected in 15–57.1% of hepatocellular carcinoma (HCC) surgical specimens, is a key risk factor for postoperative recurrence [6]. It has been suggested that MVI plays an important role in therapeutic decisions including choosing surgery or ablation, intensive postoperative monitoring and adjuvant therapies [7,8,9].

Despite the absence of microvascular invasion (MVI), a clinically significant subset of patients still experiences early recurrence (7.5% within 12 months), leading to adverse outcomes [10]. The critical, unresolved clinical question is: which specific factors drive early recurrence (ER) in this MVI-negative subgroup, and how can we accurately identify these high-risk patients. Current postoperative surveillance strategies, which involve frequent hospital visits for imaging and laboratory tests, present significant challenges in terms of cost and patient compliance. Therefore, the development of non-invasive predictive tools is crucial to identify MVI-negative hepatocellular carcinoma (HCC) patients who are at high risk of postoperative recurrence.

A variety of comprehensive scoring systems and nomogram prediction models has been used to help predict postoperative early recurrence in patients with HCC [5,11]. However, no universally recognized method for the prediction of early recurrence has been established and these models are usually constructed based on linear assumptions, which may not capture the complex relationships among different predictor variables that may exist [12]. Recently powerful AI techniques, particularly machine learning (ML) and deep learning (DL), have the capability to mine clinical insights from vast datasets, thereby facilitating informed clinical decision-making [13,14]. CatBoost, a novel machine learning algorithm, has demonstrated efficacy in developing treatment recommendations for cancer patients [15]. Furthermore, while current predictive models for hepatocellular carcinoma (HCC) recurrence primarily rely on imaging including B-scan ultrasonography and MRI (Magnetic Resonance Imaging) and histopathological data [16,17], there is a notable gap in leveraging routinely available clinical parameters through ML for MVI-negative patients. To address this gap, our study was specifically designed to develop and validate an interpretable machine learning model using accessible clinical data to predict early recurrence in MVI-negative HCC patients. We systematically benchmarked seven distinct ML algorithms to identify the optimal approach. Furthermore, by employing the SHAP interpretability framework, our research aims to identify and quantify the impact of key clinical biomarkers driving recurrence risk in this population, thereby addressing the critical need for personalized risk stratification in MVI-negative HCC management.

## 2. Materials and Methods

### 2.1. Subjects

Clinical data and postoperative follow-up information for 578 patients with primary liver cancer who underwent radical resection at Tongji Hospital, affiliated with Tongji Medical College of Huazhong University of Science and Technology, between June 2018 and January 2020, were retrospectively collected. This study received approval from the Institutional Ethics Committee of Tongji Hospital (TJ-IRB20220803).

**Figure 1:**
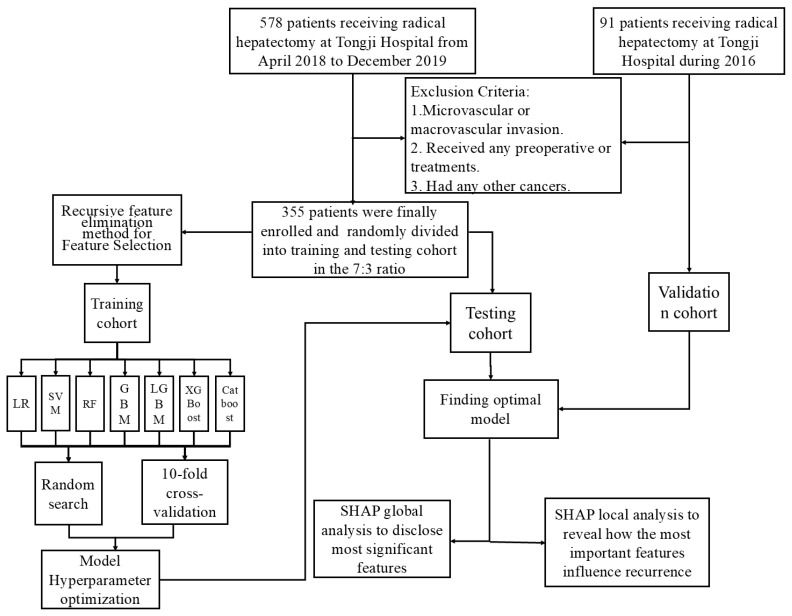
Flowchart of data processing.

The inclusion criteria were: (1) HCC without microvascular invasion confirmed by postoperative histologic examination of surgical specimens; (2) Child–Pugh class A or B liver function; (3) underwent preoperative abdominal contrast-enhanced MRI; and (4) achieved R0 tumor resection. Conversely, patients were excluded for any of the following: (1) presence of macrovascular invasion; (2) receipt of any preoperative anticancer treatments (e.g., interventional, immunologic, or targeted therapy); or (3) a history of other malignancies. Additionally, 91 patients meeting these criteria from Tongji Hospital during 2016 were selected as the external validation cohort. The patient selection process for the training and external validation groups is illustrated in Figure 1.

### 2.2. Data Collected

We collected patient characteristics such as general demographic characteristics, past medical history, laboratory tests and general imaging data of the patients. 1. Demographic characteristics, including age, sex, BMI. 2. Laboratory tests, including HBV-DNA load, HBsAg, alpha-fetoprotein (AFP) concentration, total bilirubin (TB), albumin (ALB), lymphocyte count, hemoglobin, platelet count and so on. 3. Operation-related information, including surgical approach, resection method, estimated blood loss (EBL), operation time (OT), blood transfusion. 4. Postoperative pathology information, including number of tumors, tumor size, tumor capsule, perineural invasion, satellite lesions and so on.

### 2.3. Statistical Analysis

Feature selection was performed using Recursive Feature Elimination (RFE) on the training cohort to identify the optimal feature subset. Model development and hyperparameter tuning were conducted using ten-fold cross-validation on the training set with grid search. Seven ML algorithms were evaluated: Logistic Regression (LR), Support Vector Machine (SVM), Random Forest (RF), Gradient Boosting Machines (GBM), eXtreme Gradient Boosting (XGBoost), Categorical Boosting (CatBoost), and Light Gradient Boosting Machine (LightGBM). The final optimized models were evaluated on the independent test set using metrics including the area under the receiver operating characteristic curve (AUC-ROC), accuracy, precision, recall, F1-score, calibration curves, and decision curve analysis (DCA). Model interpretability was achieved using SHapley Additive exPlanations (SHAP) analysis. Survival analysis (Kaplan–Meier curves, log-rank test) was used to assess the prognostic significance of key predictors identified by the model. Predictive modeling was implemented in Python (v3.9) using Scikit-learn (v1.2). Univariate and multivariate Cox regression analyses were performed using R software version 4.2.1. The nomogram, ROC curves, C-index, calibration curve, DCA, and survival figures were prepared or performed using R software version 4.2.1.

### 2.4. Follow-Up

The primary outcome of this study was disease-free survival (DFS). Following curative surgery, patients underwent regular follow-up assessments every 3–6 months for 2 years. During these visits, serum alpha-fetoprotein (AFP) levels were measured and imaging studies (contrast-enhanced computed tomography [CT] or magnetic resonance imaging [MRI]) were performed. Tumor recurrence was defined as either radiologically suspicious findings or biopsy-confirmed evidence of malignancy. DFS was defined as the time interval from the date of surgery to the date of tumor recurrence identification.

## 3. Results

### 3.1. Patient Characteristics

The study cohort comprised 355 patients with hepatocellular carcinoma (HCC) from Tongji Hospital, stratified into non-recurrence (*n* = 248, 69.9%) and recurrence (*n* = 107, 30.1%) groups (Table 1). Key demographic, tumor-related, and laboratory characteristics demonstrated significant heterogeneity between groups (Table 1). Compared to the non-recurrence group, recurrence patients exhibited larger maximum tumor diameter (*p* < 0.001), higher rates of tumor capsule absence (*p* < 0.001) and multiple tumor numbers (*p* < 0.001). For viral load and biomarkers, patients in the recurrence group exhibited an elevated HBV-DNA load (3120.0 vs. 177.0 IU/mL; *p* = 0.0027), higher AFP (114.2 vs. 14.04 ng/mL; *p* < 0.001) and PIVKA-II (381.0 vs. 173.0 mAU/mL; *p* = 0.0124).

### 3.2. Feature Selection in Models

**Figure 2:**
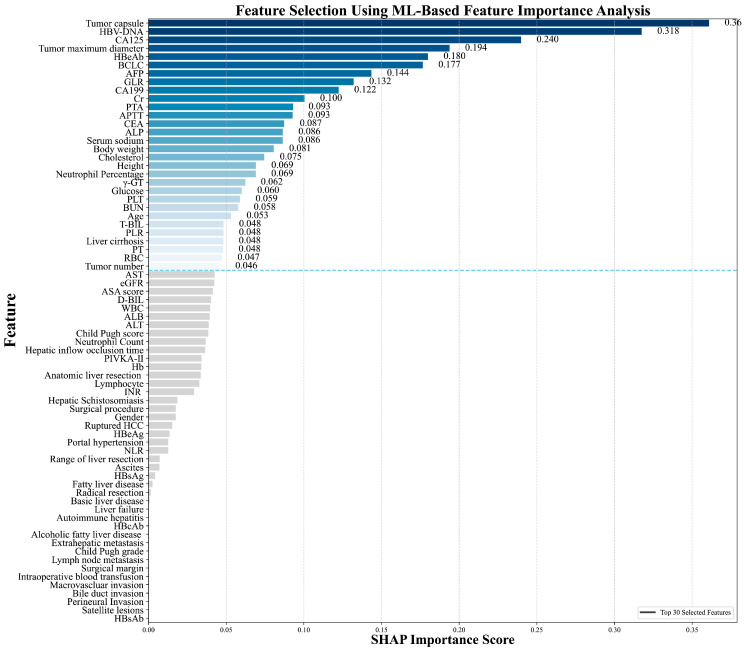
Importance of feature variables analyzed by the Recursive Feature Elimination (RFE) algorithm.

In this study, the Recursive Feature Elimination (RFE) algorithm was utilized to select features from the training cohort data. This process led to the identification of the 30 most significant features for model development. The significance of these features, ranked by importance using SHAP analysis, is presented in Figure 2.

### 3.3. Model Comparison

In the model development and validation stage, we first determined the optimal hyperparameters for each model. Prediction models were constructed based on Logistic Regression, SVM, Random Forest, GBM, XGBoost, CatBoost and LightGBM using above-mentioned 30 parameters. Specifically, for CatBoost, the optimal hyperparameters for the model were: depth = 4, iterations = 100, l2_leaf_reg = 3, and learning_rate = 0.1.

**Figure 3:**
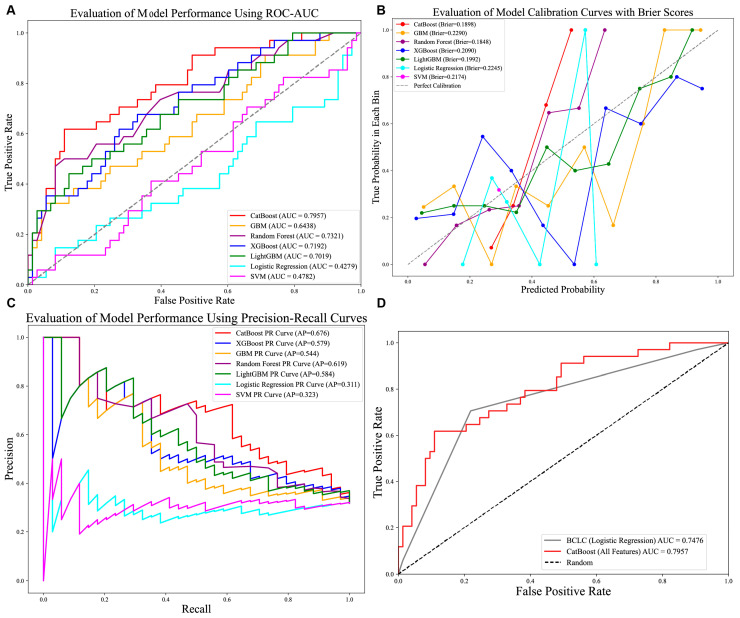
Comparison of seven prediction models. (**A**) AUROCs of seven models in internal test set. CatBoost achieved better than the other models; (**B**) Calibration performance with Brier score in seven models. (**C**) P-R curve of seven models in test set. (**D**) AUC comparison of CatBoost and standard BCLC stage system. Abbreviations: AUROCs—Area Under the Receiver Operating Characteristic Curve, P-R curve—Precision–Recall Curve.

**Figure 4:**
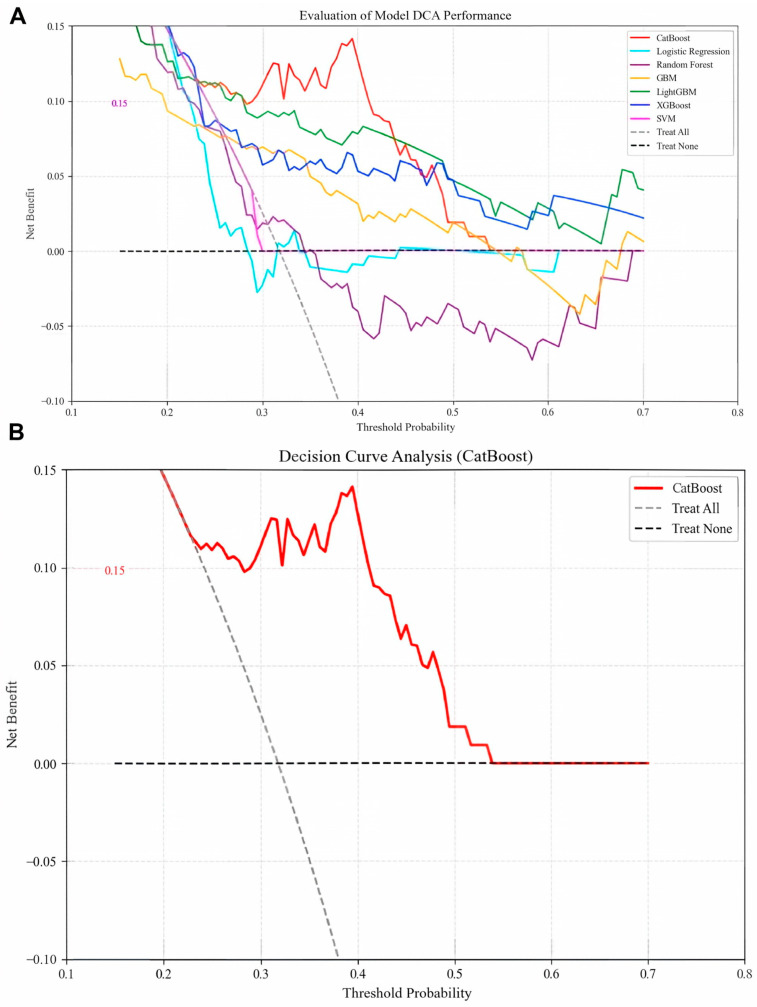
(**A**) Decision curve analysis of six machine learning models. The CatBoost model is the best diagnostic tool for early postoperative hepatocellular carcinoma recurrence. (**B**) Decision curve analysis of CatBoost model.

Optimal hyperparameters were identified for each model (e.g., CatBoost: depth = 4, iterations = 100, l2_leaf_reg = 3, learning_rate = 0.1). Performance evaluation on the test set revealed that CatBoost achieved the highest AUC (0.7957; Figure 3A), accuracy (0.7290), precision (0.7132), recall (0.7290), and F1-score (0.7123; Table 2), outperforming the other models. The CatBoost model demonstrated strong calibration performance, with a Brier score of 0.1898 (Figure 3B), alongside its high precision–recall capability (AP: 0.676; Figure 3C). Clinically, this calibration quality is critical as it ensures that the predicted probabilities reliably reflect true patient risk based on the squared difference between predicted recurrence probabilities and actual outcomes is approximately 0.19. We further compared the performance of the CatBoost model in predicting recurrence compared with the currently used BCLC staging, and we found that the CatBoost model had good performance and was superior to the BCLC stage Figure 3D. Meanwhile, the DCA curves also demonstrated good clinical utility, showing preferable positive net benefit. The DCA results revealed that the CatBoost model was the best diagnostic tool and had good clinical utility compared to other models Figure 4A,B.

### 3.4. External Validation with Time-Specific MVI-Negative HCC Dataset

We conducted external temporal validation to assess the generalization performance of our models using the time-specific HCC dataset from Tongji Hospital during 2016. The data for these years were preprocessed using the same methodology as previously applied.

**Figure 5:**
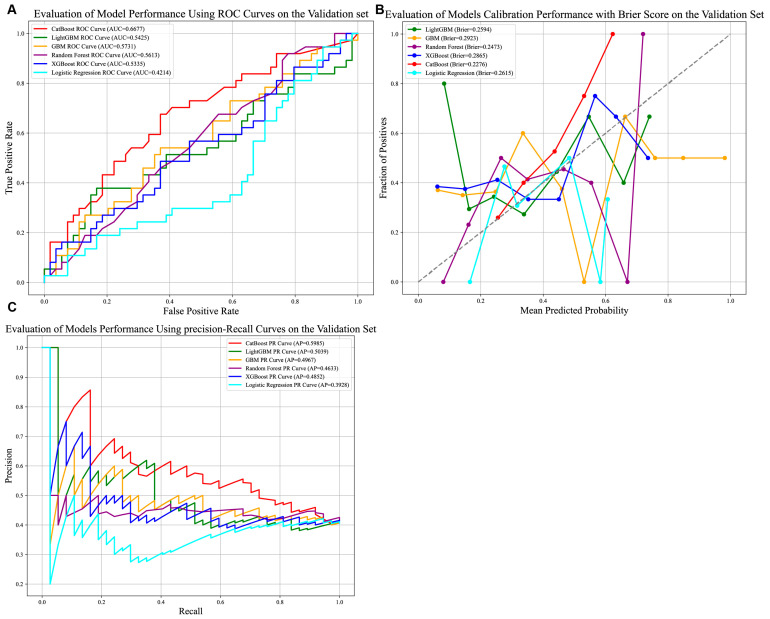
Comparison of prediction models in the external validation group. (**A**) AUROCs of six models in the external validation set. CatBoost achieved better than the other models; (**B**) Calibration performance with Brier score in six models. (**C**) P-R curve of six models in external test set. Abbreviations: AUROCs—Area Under the Receiver Operating Characteristic Curve, P-R curve—Precision–Recall Curve.

As illustrated in Figure 5A–C, the CatBoost model demonstrated the strongest discriminative ability among the six evaluated models, achieving an AUC of 0.6677. While this indicates modest predictive utility, it reflects the model’s capacity to stratify patients under evolving clinical conditions. The model further exhibited robust calibration (Brier score = 0.2276) and the highest precision–recall performance (Average Precision [AP] = 0.5985), underscoring its reliability in identifying true positive cases within the external cohort.

Clinically, the CatBoost model’s precision of 0.7081 (Supplementary Table S1) suggests a critical characteristic for avoiding unnecessary interventions. Its accuracy of 0.6264, however, highlights persistent challenges in classifying heterogeneous HCC cases, with approximately 37% of predictions misclassified. These results collectively indicate that the model offers a clinically actionable tool for risk stratification in the external validation dataset.

### 3.5. Cox Recurrence Model

Based on the selected features, we choose the top nine features to construct Cox regression model.

Multivariate Cox regression analysis showed that capsule [hazard ratio (HR) = 0.42; 95% confidence interval (CI), 0.27–0.63; *p* < 0.001], BCLC [hazard ratio (HR) = 1.85; 95% confidence interval (CI), 1.30–2.63; *p* < 0.001], CA125 (HR = 1.01; 95% CI, 1.01–1.02; *p* < 0.001), tumor size (HR = 1.06; 95% CI, 1.00–1.13; *p* = 0.043) were independent risk factors for postoperative recurrence of MVI-negative HCC patients (Supplementary Figure S1). And we use the Cox model to predict the recurrence risk based on aforementioned top nine features in MVI-negative HCC patients. To facilitate clinical application, we converted the complex mathematical model into a nomogram (Supplementary Figure S2A). Higher total scores calculated using the nomogram were related to higher risk of postoperative recurrence. The area under the ROC curve values for our nomogram for predicting the 1- and 2-year DFS were 0.779 and 0.764, respectively. The calibration curve showed that the predicted 1- and 2-year recurrence probabilities were roughly similar to the actual situation (Supplementary Figure S2C,D). Decision-curve analysis revealed that the nomogram conferred substantial net clinical benefit across clinically relevant threshold probabilities (Supplementary Figure S2E).

### 3.6. Interpretability Analysis

**Figure 6:**
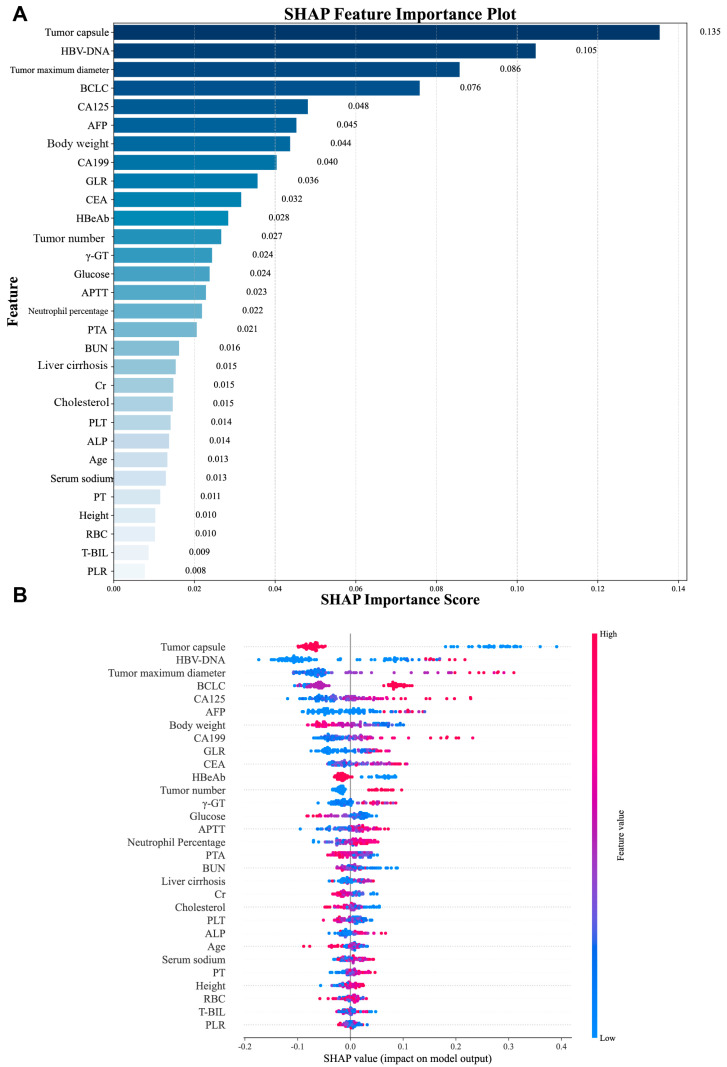
SHAP-based global interpretation of the CatBoost model. (**A**) The importance of each feature to the model using mean SHAP values, displayed in descending order. (**B**) SHAP summary dot plot, red represents larger values and blue for smaller values.

We first assessed the global interpretability of the baseline model. The CatBoost model was regarded as the baseline model as it was found to be the best performing model. Two types of SHAP plots were used to assess the model’s global interpretability: the SHAP importance plot (Figure 6A) and the SHAP summary plot (Figure 6B). SHAP feature importance analysis identified tumor capsule, HBV-DNA, tumor maximum diameter, BCLC stage, and CA125 as the top predictors influencing recurrence risk. The SHAP summary plot revealed distinct value–impact relationships: higher values of features like tumor capsule and body weight were associated with decreased recurrence risk (negative SHAP values), while lower values of HBV-DNA, CA125, and tumor maximum diameter corresponded to decreased risk (positive SHAP values). To complement this global analysis, we performed local interpretability using SHAP waterfall plots for the top 9 influential features. These plots quantify the direction (positive SHAP value increases recurrence probability, negative decreases it) and magnitude of each feature’s contribution to individual predictions.

**Figure 7:**
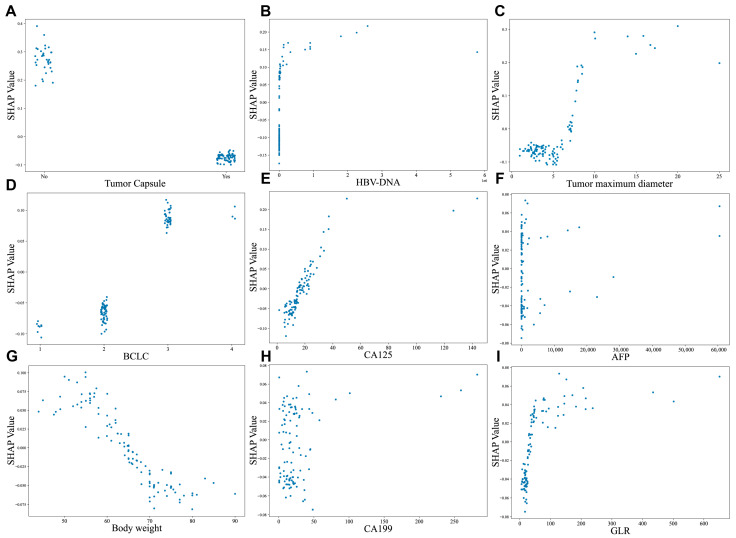
SHAP-based dependence plot of the CatBoost model. SHAP local plots for top 9 most important features. (**A**) Tumor capsule, (**B**) HBV-DNA, (**C**) Tumor maximum diameter, (**D**) BCLC, (**E**) CA125, (**F**) AFP, (**G**) Body weight, (**H**) CA199, (**I**) GLR. The *x*-axis indicates the value of certain features, while the *y*-axis shows the SHAP values of features. Higher SHAP values indicate that the feature contributes to increasing the predicted risk, while negative SHAP values suggest a contribution to reducing the predicted risk. Abbreviations: HBV-DNA—Hepatitis B virus deoxyribonucleic acid, CA125—Carbohydrate Antigen 125, BCLC—Barcelona Clinic Liver Cancer staging system, HBeAb—Hepatitis B e Antibody, GLR—Gamma-Glutamyl Transferase-to-Lymphocyte Ratio.

In Figure 7, the SHAP dependence plots further clarify the effect of variables on the CatBoost algorithm prediction. Patients with the presence of tumor capsule and decreased HBV-DNA were linked to lower SHAP values, suggestive of a lower likelihood of experiencing postoperative early recurrence (decrease in the *y*-axis) (Figure 7A,B). Moreover, increased tumor size, CA125 level, AFP, body weight, CA199, advanced BCLC stage and higher GLR were all associated with early recurrence (increase in the *y*-axis) (Figure 7C–I).

Importantly, Kaplan–Meier survival analysis independently validated the significant prognostic value of these key SHAP-identified features. Patients lacking a tumor capsule (Supplementary Figure S3A), or with high HBV-DNA, high CA125, or large tumor diameter (Supplementary Figure S3B–D) had significantly shorter progression-free survival (PFS, log-rank *p* < 0.001). Conversely, higher body weight was associated with improved PFS (Supplementary Figure S3E).

Individualized predictions were visualized using SHAP force plots, illustrating feature contributions to model outputs for specific patients (representative examples are shown in Supplementary Figure S4A–D).

## 4. Discussion

While radical liver resection improves survival in HCC patients, early recurrence (ER) remains a major challenge impacting outcomes [18]. Early detection of ER and the application of precise treatments can markedly improve prognosis and survival in HCC patients. Therefore, developing prediction models for ER is crucial. Traditionally, Cox regression models have been used to predict HCC recurrence, demonstrating good diagnostic efficacy. However, these existing models, including those based on Cox regression [19], or staging systems like BCLC [20,21], often rely on linear assumptions and may overlook critical biomarkers such as HBV-DNA and CA125, thus limiting their predictive power for ER, particularly in subgroups like MVI-negative HCC. Recent studies employing machine learning (ML) show promise in capturing complex interactions for recurrence prediction. Recently, Liu et al. [5] conducted a Hemoglobin, Albumin, Lymphocyte, and Platelet (HALP) score-based nomogram for predicting early recurrence in BCLCstage 0/A HCC patients with a 0.756 AUC, which had limited predictive effect. In contrast, machine learning approaches, particularly when integrating diverse medical data sources and algorithmic frameworks, have demonstrated superior capability in modeling these intricate relationships, thereby increasingly supplanting conventional regression methods. For example, Zhang et al. selected XGBoost as the optimal model, achieving excellent predictive efficiency with an optimal cut-off value of 55% for the prediction probability [22].

In this study, we developed and validated a machine learning model based on CatBoost specifically tailored to predict early recurrence in microvascular invasion (MVI)-negative hepatocellular carcinoma (HCC) patients after curative resection. Our model demonstrated robust predictive performance. When compared to other models, CatBoost significantly outperformed its counterparts, achieving an area under the curve (AUC) of 0.7957 (Figure 3A). This performance was approximately 0.06 higher than the second-best model, Random Forest, which had an AUC of 0.7321. CatBoost’s performance also exceeded that of the weaker-performing models, LightGBM (AUC: 0.7019) and XGBoost (AUC: 0.6869). These differences hold practical significance in clinical decision-making, particularly in scenarios requiring high discriminative power. Furthermore, the CatBoost model demonstrated excellent calibration, evidenced by the close alignment of predicted probabilities with observed recurrence rates across the risk spectrum (Figure 3B) and quantified by a low Brier score of 0.1898. A Brier score of 0.1898 indicates that, on average, there is a squared difference of approximately 0.19 between the predicted recurrence probabilities and actual outcomes (1 for recurrence, 0 for no recurrence). This level of calibration accuracy is clinically significant because it means the model’s predicted probabilities reliably reflect the true underlying risk of recurrence for individual patients. However, there may be slight deviations in the calibration curve where predicted probabilities are close to 0.5, requiring further validation in a larger sample.

CatBoost also demonstrated superior performance with an average precision (AP = 0.676) compared to other models (AP = 0.584 or lower), which is consistent with its advantage in ROC-AUC (Figure 3C). Furthermore, SHapley Additive exPlanations (SHAP) analysis was employed to identify the most influential predictive features from the clinical variable pool and to ensure model interpretability. Overall, we developed an effective machine learning model, which showed good predictive performance. The SHAP force plots visualize the contribution of each feature to the model prediction of ER in each individual. Figure 3D clearly quantifies the predictive superiority of the CatBoost model over the current clinical BCLC staging system. Specifically, CatBoost achieved an improvement in AUC compared to the BCLC staging. This finding holds significant clinical implications, indicating that machine learning-based models have the potential to outperform traditional staging systems and provide more individualized and precise predictions of recurrence risk. However, the BCLC staging system, as a widely validated prognostic tool, possesses the simplicity and clinical interpretability that complex models often lack. Future prospective studies should validate whether this translates to improved clinical outcomes, given BCLC’s established simplicity and interpretability.

Tumor capsule, HBV-DNA, tumor maximum diameter, BCLC stage and CA125 were considered to be the five most important factors. Among them, the tumor capsule was the most important factor. The tumor capsule has garnered extensive utilization in clinical surveys concerning HCC for the assessment of postoperative recurrence. A research endeavor spearheaded by Zhang et al. [23] sought to authenticate the association between tumor capsule and prognosis in HCC patients after hepatectomy. The study outcomes unveiled a significant association between incomplete tumor capsule and postoperative recurrence, consistent with another study [24]. Biologically, an intact tumor capsule serves as a physical barrier to restrict cancer cell dissemination. Our study echoes this by demonstrating a correlation between absence of tumor capsule and recurrence after hepatectomy, aligning with prior research.

Our study further identified elevated serum HBV DNA levels as a significant predictor of increased HCC recurrence risk. As an established biomarker for monitoring HBV activity in chronic infection [25], high HBV DNA reflects uncontrolled viral replication in the background liver—a major risk factor for hepatocarcinogenesis. Uncontrolled HBV infection within the liver significantly contributes to HCC development. Moreover, HBV DNA level has been proved to be a risk predictor associated with both early recurrence and late recurrence [26]. Mechanistically, as described by Kobayashi S et al. [27], HBV infection triggers an inflammatory response in Kupffer cells, leading to cytokine and chemokine production, which promotes HCC development and recurrence. This virus-induced inflammatory microenvironment fosters HCC cell survival and proliferation [28,29].

Additionally, tumor size has been identified as a significant predictor of early HCC recurrence, consistent with established evidence that larger tumors correlate with poorer prognosis after curative hepatectomy [30,31]. Furthermore, our findings also reveal a positive correlation between BCLC staging and early HCC recurrence, reinforcing its prognostic relevance.

Elevated tumor biomarkers are independently associated with increased tumor burden, metastatic potential, adverse prognosis, and postoperative recurrence in hepatocellular carcinoma (HCC). While serum alpha-fetoprotein (AFP) is elevated in approximately two-thirds of HCC patients and remains the most widely utilized diagnostic marker, [32] the prognostic significance of CA125 has been less explored. Huang et al. [33] demonstrated that preoperative CA125 elevation predicts reduced recurrence-free (RFS) and overall survival (OS) after curative hepatectomy, highlighting the need for intensified surveillance and adjuvant therapy in this high-risk subgroup. Similarly, another study indicated that elevated preoperative serum CA125 concentrations (>30 U/mL) were associated with poor prognosis of patients and larger tumor diameter which is apparently related to HCC recurrence [34]. Our data further establish CA125 as a potential early biomarker for recurrent disease. Mechanistically, MUC16/CA125 promotes immune evasion in multiple malignancies by suppressing natural killer (NK) and T-cell function via tumor-associated regulatory T cells (Tregs) [35], and MUC16/CA125 has been shown to suppress antitumor immune responses by inhibiting the function of natural killer (NK) cells and T cells. A similar mechanism might be at play in hepatocellular carcinoma (HCC) [36,37,38].

Additionally, tumor size has been identified as a significant predictor of early HCC recurrence, consistent with established evidence that larger tumors correlate with poorer prognosis after curative hepatectomy [37,38]. Furthermore, our findings also reveal a positive correlation between BCLC staging and early HCC recurrence, reinforcing its prognostic relevance.

A European cohort study has confirmed obesity as a well-established risk factor for multiple malignancies. Notably, it found significant increases in the risk of endometrial cancer, renal cell carcinoma in men, and colorectal cancer in males [39]. However, it remains controversial that obesity contributes to HCC prognosis and recurrence according to studies. Ji et al. [40] reported that compared to patients with high BMI (>18.5), those lower exhibited shorter DFS and OS. This association may be attributed to the role of body mass index (BMI) as an immunonutritional indicator. Reduced BMI, often reflecting malnutrition, typically leads to immunosuppression. Supporting this mechanism, a study examining obesity and hepatocellular carcinoma (HCC) recurrence after liver transplantation identified obesity as an independent predictor of earlier tumor recurrence [41]. Conversely in a cohort of 427 adult recipients transplanted for HCC [42], the recipient’s BMI at the time of liver transplantation and during subsequent follow-up did not significantly influence the recurrence of hepatocellular carcinoma or the long-term survival rates of the patients. This consistency persisted across subgroups stratified by baseline characteristics. However, our study demonstrated a protective effect of higher body weight against early recurrence, aligning with the findings reported by Ji et al. [40].

It is worth noting that some of the features identified by our ML model as key predictors of early recurrence are also part of the traditional diagnostic criteria used in current clinical protocols. For instance, tumor capsule integrity and tumor size are factors commonly assessed in pathological examinations and are known to influence HCC recurrence risk [24,43]. Similarly, serum AFP levels are a conventional biomarker for HCC diagnosis and prognosis [44]. However, our study goes beyond these traditional criteria by incorporating additional variables such as HBV-DNA, CA125, and body weight into the predictive model. This expanded set of features allows for a more comprehensive assessment of recurrence risk. The ML model, particularly the CatBoost model, effectively integrates these diverse variables and their complex interactions, providing a more accurate prediction of early recurrence compared to models based solely on traditional diagnostic criteria. The traditional diagnostic criteria are crucial for initial diagnosis and prognosis assessment, but they may not capture the full spectrum of factors contributing to early recurrence. Compared with them, our CatBoost model is designed to be seamlessly integrated into existing clinical workflows. It utilizes routinely available preoperative and postoperative parameters—such as tumor capsule status, HBV-DNA, CA125, tumor size, and body weight—which are readily accessible from electronic medical records (EMR) and laboratory systems. The model can be deployed as a web-based tool or embedded within hospital information systems (HIS) to provide real-time, individualized recurrence risk assessments. Moreover, clinicians can input patient data to receive a recurrence probability score accompanied by a SHAP force plot, which visually explains the contribution of each feature to the prediction. This output can guide postoperative management strategies, such as intensifying surveillance for high-risk patients or considering adjuvant therapies, enables the development of more personalized and targeted treatment strategies, potentially improving patient outcomes.

## 5. Limitations

While our study presents a clinically interpretable CatBoost model for predicting early recurrence (ER) in MVI-negative HCC, several limitations warrant acknowledgment: Firstly, although the CatBoost model achieved robust performance in the internal test set (AUC: 0.7957; Accuracy: 0.7290; Table 2), its external validation on a temporal cohort (*n* = 91) revealed moderated metrics (Accuracy: 0.6264; Precision: 0.7081; Recall: 0.5448; AUC: 0.6677; Supplementary Table S1). These suggests that the model’s performance moderated upon external temporal validation, highlighting a critical boundary in its current generalizability. This variability underscores that our model is not yet ready for immediate broad clinical implementation without further refinement and validation. Secondly, the model was developed on a single-center retrospective cohort with a limited sample size and event rate. This boundary necessitates caution in interpreting the feature importance and absolute performance metrics, as they may be susceptible to instability. To transcend this limitation, we plan to initiate a prospective, multi-center data collection effort. This will not only expand the dataset to improve model power and reduce overfitting but also allow us to investigate the integration of more novel biomarkers which could further boost predictive accuracy. Looking further ahead, our ultimate goal is clinical translation. Therefore, following successful external validation, we plan to design a prospective clinical trial. The trial will evaluate the efficacy of the model in guiding clinical decision-making compared to standard care. The primary endpoints of such a trial would be improvements in recurrence-free survival and overall survival, providing the highest level of evidence for the model’s clinical utility.

## 6. Conclusions

In conclusion, this study leveraged machine learning techniques to develop a predictive model based on machine learning algorithms for early recurrence in HCC patients without microvascular invasion (MVI). By analyzing a comprehensive set of clinical and laboratory parameters, we established a robust framework using the CatBoost algorithm, which demonstrated superior performance in predicting early recurrence, achieving 0.7957 AUC and 0.7290 accuracy. The integration of SHAP analysis not only enhanced the interpretability of the model but also identified critical biomarkers such as tumor capsule absence, elevated HBV-DNA, CA125 levels and tumor diameter, and lower body weight as key predictors of recurrence risk. These findings are consistent with existing literature and provide deeper insights into the clinical factors influencing HCC recurrence. By leveraging existing data sources and providing interpretable outputs, our model supports clinical decision-making without requiring additional diagnostic tests, thereby enhancing its practicality and adoption potential in real-world settings.

These visualizations may aid clinicians in identifying high-risk patients who could benefit from intensified surveillance or adjuvant therapy discussions. By bridging the gap between complex machine learning algorithms and clinical practice, our study provides a transparent and actionable tool for managing HCC patients in real-world settings.

## Figures and Tables

**Table 1:** Baseline characteristics of HCC patients without MVI from Tongji Hospital (*n* = 355).

Feature	Non-Recurrence	Recurrence	*p*-Value
	(*n* = 248)	(*n* = 107)	
Age, mean (SD)	55.0 (15.0)	53.0 (14.0)	0.0341
Tumor maximum diameter	4.40 (3.10)	6.10 (5.20)	0
CA125	14.15 (9.77)	16.90 (12.37)	0.0001
GLR	26.84 (30.37)	45.30 (59.36)	0.0001
AFP	14.04 (342.78)	114.20 (1657.60)	0.0001
AST	26.00 (12.00)	31.00 (19.50)	0.0001
γ-GT	41.00 (43.25)	56.00 (84.00)	0.0005
HBV-DNA	177.00 (24,050.00)	3120.00 (167,400.00)	0.0027
ALB	41.95 (5.67)	40.60 (5.60)	0.0037
Cr	76.00 (16.25)	70.00 (19.50)	0.008
PIVKA-II	173.00 (1212.75)	381.00 (2752.50)	0.0124
eGFR	94.60 (15.53)	96.00 (15.10)	0.0214
ALP	76.00 (30.25)	81.00 (46.00)	0.0658
D-BIL	4.70 (2.83)	5.30 (3.05)	0.0705
Neutrophil Percentage	58.40 (13.07)	60.70 (11.90)	0.1124
Blood glucose	5.19 (1.04)	5.08 (0.94)	0.115
INR	1.06 (0.11)	1.07 (0.10)	0.138
BUN	5.40 (1.71)	5.10 (1.91)	0.1496
CA199	15.36 (16.42)	16.73 (19.27)	0.1722
Serum sodium	140.95 (2.50)	141.00 (2.40)	0.1904
Cholesterol	3.69 (1.10)	3.58 (1.12)	0.2329
Neutrophil Count	2.88 (1.66)	3.12 (1.36)	0.2434
CEA	2.73 (1.15)	2.67 (1.42)	0.4103
Body weight	65.90 ± 8.84	63.60 ± 9.05	0.0258
PTA	90.76 ± 11.55	88.93 ± 10.25	0.1596
APTT	38.28 ± 3.74	38.76 ± 3.69	0.2675
Gender, *n*			
Male	220	89	0.2106
Female	28	18	
Tumor capsule, *n*			
Absence	41	44	0
Presence	207	63	
BCLC, *n*			0
0	23	2	
A	146	34	
B	76	65	
C	3	6	
HBeAb, *n*			0.1299
Negative	56	33	
Positive	192	74	
Tumor number, *n*			0
Single	210	59	
Multiple	38	48	
ASA score, *n*			0.9222
1	17	8	
2	164	70	
3	66	29	
4	1	0	

**Table 2:** Comparison of the performance of seven ML models in the training cohort. Abbreviations: LR—Logistic Regression, SVM—Support Vector Machine, GBM—Gradient Boosting Machines, XGBoost—eXtreme Gradient Boosting, LightGBM—Light Gradient Boosting Machine.

Model	Accuracy	Precision	Recall	F1-Score	AUC Score
LR	0.6822	0.6267	0.6822	0.5696	0.4279
SVM	0.6822	0.4655	0.6822	0.5534	0.4782
Random Forest	0.7196	0.7336	0.5745	0.5556	0.7321
GBM	0.7009	0.6790	0.7009	0.6802	0.6438
XGBoost	0.7290	0.7208	0.7290	0.6855	0.6869
CatBoost	0.7290	0.7132	0.7290	0.7123	0.7957
LightGBM	0.7009	0.7921	0.7009	0.5949	0.7019

## Data Availability

The datasets utilized and/or analyzed in this study are available from the corresponding author upon reasonable request.

## Supplementary Materials

The following supporting information can be downloaded at: https://www.mdpi.com/article/10.3390/biomedicines13102507/s1.

## Abbreviations

The following abbreviations are used in this manuscript:

HCC	Hepatocellular carcinoma
MVI	Microvascular invasion
ER	Early recurrence
ML	Machine learning
DL	Deep learning
RFE	Recursive Feature Elimination
SHAP	SHapley Additive exPlanations
AUC	Area Under the Curve
ROC	Receiver Operating Characteristic
LR	Logistic Regression
SVM	Support Vector Machine
RF	Random Forest
GBM	Gradient Boosting Machines
XGBoost	eXtreme Gradient Boosting
LightGBM	Light Gradient Boosting Machine
CatBoost	Categorical Boosting
DFS	Disease-free survival
MRI	Magnetic Resonance Imaging
CT	Computed Tomography
AFP	Alpha-fetoprotein
TB	Total bilirubin
ALB	Albumin
EBL	Estimated blood loss
OT	Operation time
IQR	Interquartile range
SD	Standard deviation
AP	Average Precision
PFS	Progression-free survival
HBV	Hepatitis B Virus
HBsAg	Hepatitis B surface antigen
CA125	Cancer Antigen 125
CA199	Carbohydrate Antigen 19-9
GLR	Gamma-glutamyl transferase to lymphocyte ratio
AST	Aspartate Aminotransferase
Cr	Creatinine
APTT	Activated Partial Thromboplastin Time
ALP	Alkaline Phosphatase
BUN	Blood Urea Nitrogen
γ-GT	Gamma-glutamyl transferase
INR	International Normalized Ratio
D-BIL	Direct Bilirubin
CEA	Carcinoembryonic Antigen
PIVKA-II	Protein Induced by Vitamin K Absence or Antagonist-II
eGFR	Estimated Glomerular Filtration Rate
PTA	Prothrombin Time Activity
BCLC	Barcelona Clinic Liver Cancer
HBeAb	Hepatitis B e antibody
ASA	American Society of Anesthesiologists
NK cells	Natural Killer cells
Tregs	Regulatory T cells
MUC16	Mucin 16
OS	Overall Survival
RFS	Recurrence-free Survival
BMI	Body Mass Index
