# Supplementary material for: Machine Learning-Based Models for the Prediction of Postoperative Recurrence Risk in MVI-Negative HCC
Source: Biomedicines. 2025 Oct 15;13(10):2507. doi: 10.3390/biomedicines13102507 (PMC12561097; doi:10.3390/biomedicines13102507)
Supplement: Supplementary file 1 [file biomedicines-13-02507-s001.zip › Supplementary Figure.pdf]

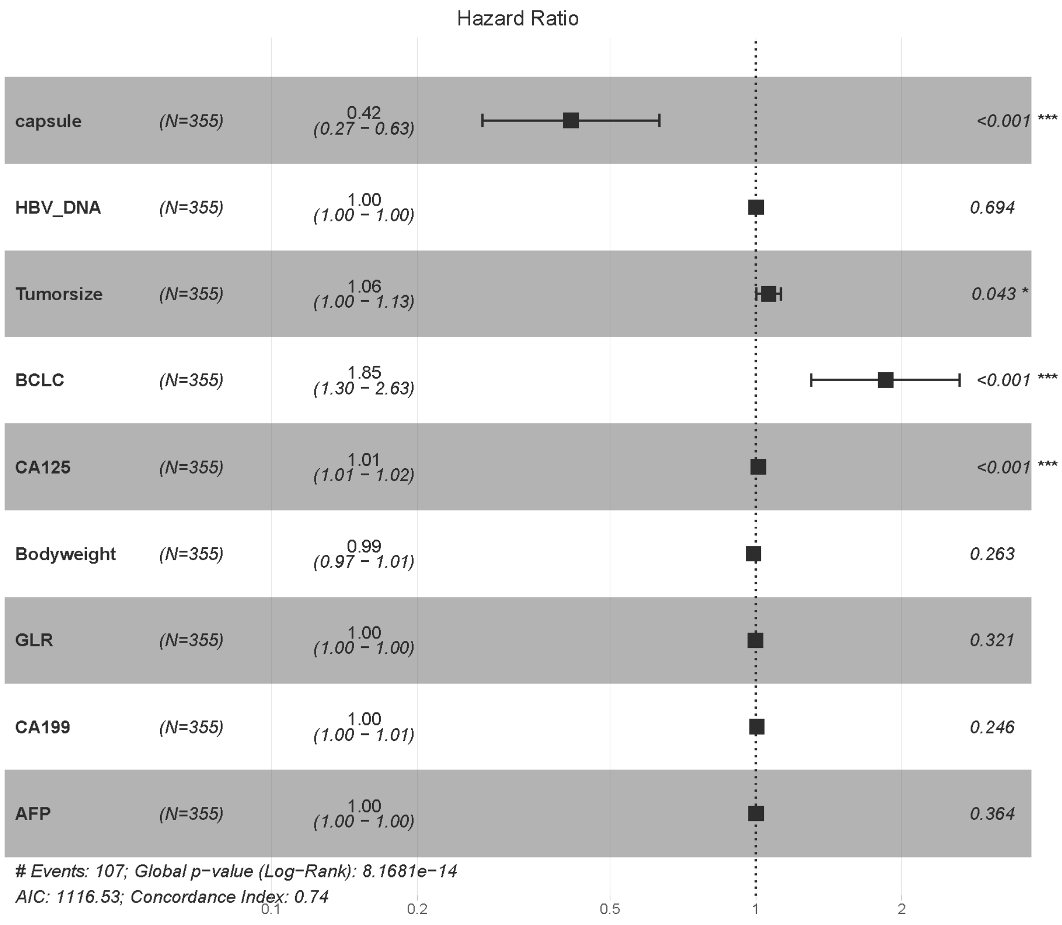

Supplementary Figure S1. Cox regression model was further established based on the top 9 parameters screened by RFE algorithm.

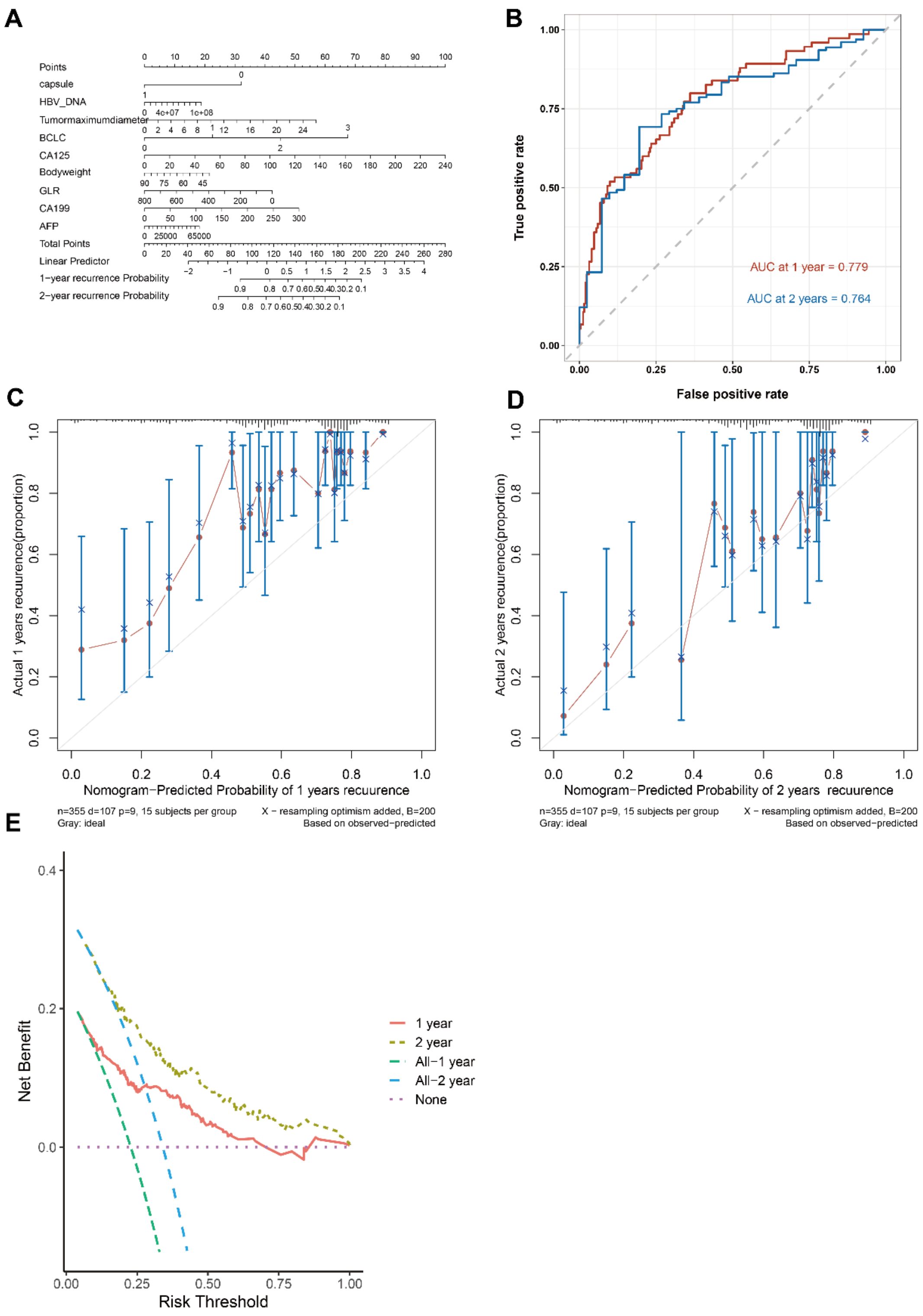

Supplementary Figure S2. A. Nomogram used to predict time-related recurrence in patients with HCC. **B. The** 1-year and 2-year ROC curve of DFS in the training cohort.

C-D. Calibration plots of 1-,2-year DFS based on Cox regression model in the training cohort. **E.** DCA plots for the nomogram in predicting the 1-,2-year DFS.

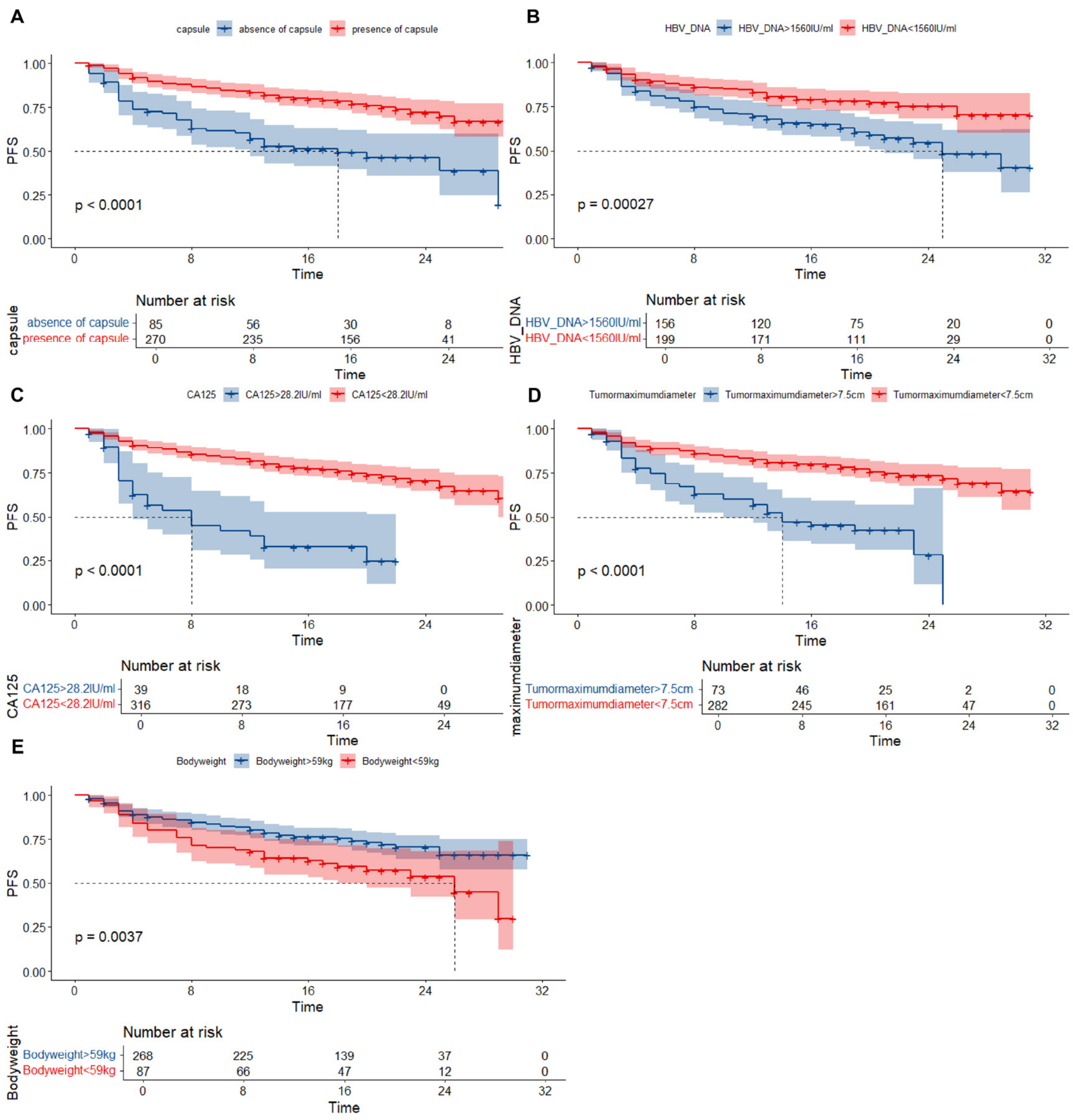

Supplementary Figure S3. Kaplan-Meier plots. (**A–E**) Kaplan–Meier plot of progression-free survival (PFS) based on Tumor capsule, HBV-DNA, CA125 concentration, Tumor maximum diameter and Body weight.

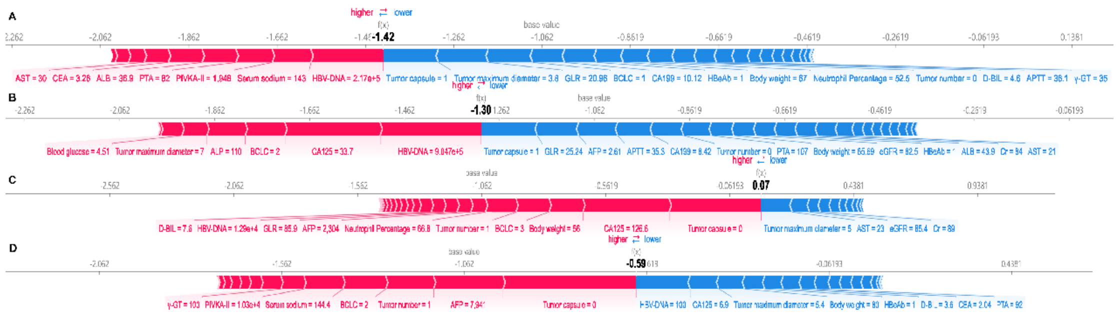

Supplementary Figure S4. Representative SHAP force plots for individual model predictions. A, B, C and D represent four randomly selected patients from the test set, illustrating the contribution of each feature to the prediction outcomes. The SHAP values indicate the prediction-related feature of individual patients and the contribution of each feature to the early recurrence. The bold-faced numbers are the probabilistic predicted values (f(x)), whereas the base values are the values predicted without giving input to the model. The f(x) is the log odds ratio of each observation, The red features (on the left) indicate features that increase the recurrence risk and the blue features indicate features that decrease the recurrence risk, The length of the arrows helps visualize the magnitude of the effect on the prediction. The longer the arrow, the larger the effect.
