# Supplementary material for: Machine Learning-Based Models for the Prediction of Postoperative Recurrence Risk in MVI-Negative HCC
Source: Biomedicines. 2025 Oct 15;13(10):2507. doi: 10.3390/biomedicines13102507 (PMC12561097; doi:10.3390/biomedicines13102507)
Supplement: Supplementary file 1 [file biomedicines-13-02507-s001.zip › Supplementary Table.pdf]

Supplementary Table S1. Comparison of the performance of ML models in the external validation cohort.

Model	Accuracy	Precision	Recall	F1-Score	AUC Score
LR	0.5714	0.4181	0.4857	0.3861	0.4214
Random Forest	0.5824	0.5119	0.5035	0.4305	0.5613
GBM	0.5934	0.5599	0.5425	0.5274	0.5731
XGBoost	0.6086	0.6443	0.5533	0.5054	0.5335
CatBoost	0.6264	0.7081	0.5448	0.4738	0.6677
LightGBM	0.6138	0.6117	0.5703	0.5524	0.5425
